# The impact of the tumor microenvironment on macrophages

**DOI:** 10.3389/fimmu.2025.1572764

**Published:** 2025-05-16

**Authors:** Man Xiao, Xiaoduan Li

**Affiliations:** Department of Obstetrics and Gynecology, Union Hospital, Tongji Medical College, Huazhong University of Science and Technology, Wuhan, China

**Keywords:** tumor microenvironment, macrophages, recruitment, reprogramming, functional modulation

## Abstract

The tumor microenvironment (TME), which has crucial roles in tumor progression, metastasis, and drug resistance, contains abundant immune cells. The most influential of these include tumor-associated macrophages (TAMs), which both secrete microenvironment-modifying cytokines and are acted upon by various other components of the microenvironment. The heterogeneity and diversity of TAMs are closely associated with patients’ response to tumor immunotherapy; thus, therapeutic targeting of TAMs has become a research focus in recent years. Although numerous studies have explored how TAMs alter the microenvironment, relatively few have investigated the impact of the microenvironment on TAMs. In this review, we discuss the effects of various components of the tumor microenvironment on TAMs from the perspectives of recruitment, reprogramming, and functional modulation, with a focus on the cellular components of the microenvironment. We also summarize the development of immunotherapies targeting TAMs, which have shown promising results in clinical trials.

## Introduction

Tumors represent a significant public health concern, with extremely high incidence and mortality rates globally (1, 2). Traditional anti-tumor therapies such as radiotherapy and chemotherapy target malignant cells directly but show limited efficacy. By contrast, immunotherapies, which act by stimulating immune cells, have the potential to elicit persistent responses; however, they are efficacious in only a small proportion of patients owing to the profound immunosuppressive effect exerted by the tumor microenvironment (TME) and the complex interactions between the tumor and the TME that occur at every stage of cancer progression, from tumorigenesis, progression, invasion, and vascular internalization to metastasis, diffusion, and growth (3, 4).

The cellular composition and functional status of the TME can differ significantly depending on the site at which the tumor develops, the intrinsic characteristics of the cancer cells, the tumor stage, and the patient’s characteristics. Generally, the TME is classified into cellular and non-cellular components. The cellular components include diverse immune cells (myeloid immune cells and lymphocytes) and stromal cells (fibroblasts, endothelial cells, and pericytes), whereas the non-cellular components comprise the extracellular matrix (ECM), as well as soluble factors (such as cytokines and chemokines), metabolites, extracellular vesicles (EVs), microRNAs (miRNAs), etc. (5, 6). The ECM is a complex network that contains collagen, non-collagen proteins (such as fibronectin), elastin, proteoglycans, and glycosaminoglycans (such as hyaluronic acid) (7). Tumor-associated macrophages (TAMs) form a significant component of the TME, accounting for up to 50% in cases of certain solid tumors, and have important roles in tumorigenesis, development, metastasis, and drug resistance (8). TAMs also play a key part in the creation of an immunosuppressive TME by generating cytokines, chemokines, and metabolites, and by recruiting and maintaining immunosuppressive cells (9–11).

There is extensive communication between TAMs and other immune components, including cytotoxic T cells, regulatory T cells, cancer-associated fibroblasts (CAFs), and neutrophils (12). Moreover, TAMs regulate the anti-tumor responses of CD8+ T cells, proliferation of regulatory T cells, activation of natural killer (NK) cells, formation of CAFs, and so on (13–17). Thus, an in-depth understanding of the interactions between TAMs and other cells in the TME is essential to the development of tumor treatment regimens (18). To date, immune-targeted therapeutics have mainly been focused on T cells and T-cell-related immune checkpoints, such as anti-programmed cell death protein 1 (PD-1)/programmed cell death ligand 1 (PD-L1) and chimeric antigen receptor T cell (CAR-T)-based therapies. However, therapies using CAR-T have not proven successful against solid tumors, possibly owing to the inhibitory nature of the TME (19). Given the limited efficacy of T cells in this context, targeting of TAMs has emerged as a potential alternative approach.

Efforts to optimize anti-cancer therapies has been accompanied by improvements in our understanding of the interactions among TME cells (4). For instance, research in glioblastoma (GBM) has shown that PD-L1 blockade combined with a dendritic cell vaccine can deplete PD-L1+ macrophages, suppress myeloid inflammation, and enhance effector T cells, leading to significant disease regression (20). Another study showed that phytosomal curcumin exerts its therapeutic effect in GBM by inducing the release of monocyte chemoattractant protein-1 (MCP-1, also known as C-C motif chemokine ligand 2 (CCL2)), from TAMs, which in turn recruits activated NK cells to GBM, resulting in anti-tumor effects (21). In addition, the selective expression of membrane spanning four domains A4A (MS4A4A) by TAMs has been reported to be associated with poor clinical outcomes in cancer patients, and anti-MS4A4A in combination with anti-PD-1 treatment has shown effectiveness for large, refractory colorectal cancer (CRC), with further combination with radiotherapy resulting in complete regression (22). These findings indicate the importance of multi-modal immunotherapy and the potential need for future immunotherapy strategies to incorporate TAM-targeting approaches to achieve maximal clinical benefits.

Given the complex network of interactions that occurs in the TME, further elucidation of the bidirectional effects between TAMs and other TME cell types is clearly important to the successful development of new targeted cancer therapies (23). However, despite numerous studies having focused on the impact of TAMs on the microenvironment, there has been relatively little research examining the influence of the TME on TAMs. In the present review, we discuss the existing literature on how various components of the TME affect TAMs from the perspectives of recruitment, reprogramming, and functional modulation, as well as considering the evaluation of relevant therapies in clinical trials.

## Origins and classification of macrophages

The developmental origin of a cell type serves as the foundation of its function. Previous studies have confirmed that macrophages originate from hematopoietic stem cells in the bone marrow and subsequently differentiate from monocytes. Advances in single-cell RNA sequencing (scRNA-seq) have enabled further elucidation of their developmental trajectory. As well as monocyte-derived macrophages (MDMs), there are specific tissue-resident macrophages (TRMs) (24), which originate from the embryonic yolk sac and fetal liver and maintain themselves through local proliferation in adulthood (25, 26).

MDMs serve as a reservoir for recruitment of additional macrophages and are mobilized in pathological conditions. Expansion of macrophages and recruitment of new monocytes within tissues are essential processes in the progression of various solid tumors (27). Moreover, both MDMs recruitment and increased expansion of TRMs occur in the TME. For instance, tissue-resident perivascular macrophages and MDMs have been found simultaneously in mouse lung cancer, and MDMs account for 85% of total TAMs in GBM (28, 29). Owing to their different developmental origins, MDMs and TRMs have distinct cytokine response patterns. For instance, colony stimulating factor 1 (CSF1)/colony stimulating factor 1 receptor (CSF1R) promotes recruitment, survival, and proliferation of MDMs and induces their polarization, whereas TRMs have a relatively low dependence on CSF1 (30). MDMs are more susceptible to regulation by the TME, whereas TRMs are already present in the tissue before tumor occurrence and show relatively limited responses to the TME (24, 31). Recruitment of macrophages usually means the process of migration from the blood circulation to the TME, and it generally refers to MDMs.

TAMs undergo reprogramming, defined as modulation of their phenotypes, particularly their polarization into specific types. Mills et al. linked T-cell-macrophage interactions with functional phenotypes, providing a theoretical basis for subsequent M1/M2 classification (32), and the Mantovani team proposed the nomenclature system of classically activated (M1, pro-inflammatory) and alternatively activated (M2, anti-inflammatory/repair) macrophages; they classified TAMs as having M2-like phenotypes (33, 34). M1 macrophages can be induced by interferon (IFN)-γ secreted by Th1 cells, and they secretes pro-inflammatory factors interleukin (IL)-1β and IL-6 and releases nitric oxide. M2 macrophages are induced by IL-4 and IL-13 secreted by T helper 2 (Th2) cells, and they secrete anti-inflammatory factors IL-10 and transforming growth factor-β (TGF-β)and express arginase-1 (Arg1) to promote tissue repair. Subsequent studies have expanded the classification of the M2 type to include four subtypes: M2a, M2b, M2c, and M2d. M2a macrophages are induced by IL-4/IL-13 and express CD206, secreting IL-10, TGF-β, and Arg1, and participate in parasitic immunity and tissue repair. The M2b types are activated by synergistic effects of immune complexes and toll-like receptor (TLR)/IL-1R ligands; they have immunoregulatory functions and express IL-10, CCL1, and CD86. The M2c types are induced by IL-10 or glucocorticoids, express CD163, participate in immunosuppression and matrix remodeling, and secrete IL-10 and matrix metalloproteinases (MMPs). Finally, M2d describes a pro-angiogenic and tumor-promoting phenotype that is activated by adenosine receptors or TLR antagonists and expresses vascular endothelial growth factor (VEGF) and IL-10 (34–36). This subtype classification of M2 reflects the high plasticity of macrophages in terms of function and the dynamic balance in which they participate regarding inflammation, repair and immune regulation. As well as secreting different cytokines, macrophages of different types show differences related to the metabolic microenvironment; M1 macrophages typically rely on glycolysis, whereas those of the M2 type are more dependent on oxidative phosphorylation (37). Metabolites of macrophages including lactate, succinate, and arginine are important non-cellular components of the microenvironment and regulate macrophage function in turn (38). For example, lactate, which is produced in large quantities by M1 macrophages, promotes tumor angiogenesis via hypoxia inducible factor 1α (HIF-1α) and upregulates VEGF and Arg1, thereby promoting M2 polarization (39, 40). Moreover, α-ketoglutarate produced by the breakdown of glutamine is crucial for alternative (M2) activation of macrophages (41).

However, despite its predominance in studies of the biology of TAMs, the M1/M2 classification scheme can be considered to be an over-simplifications that fails to adequately describe the diversity of TAM states. Therefore, Mosser and Edwards proposed the ‘color wheel’ model of macrophage function, in which macrophage phenotypes represent a dynamic continuum regulated by a combination of microenvironmental signals. This model has promoted a more in-depth understanding of macrophage heterogeneity (42). and advances in scRNA-seq technology have led to classification of TAMs according to molecular markers, functional states, spatial distribution, and metabolic characteristics (18). For instance, a study analyzing scRNA-seq data of myeloid cells from 380 samples representing 15 cancer types identified seven TAM subgroups: inhibin beta A (INHBA)+, complement C1q C chain (C1QC)+, interferon-stimulated gene 15 (ISG15)+, NLR Family Pyrin Domain Containing 3 (LNRP3)+, lymphatic vessel endothelial receptor-1 (LYVE1)+, and secreted phosphoprotein 1 (SPP1)+ TAMs (43). This classification was based on specific molecular expression, indicating that the TAM subtypes may be closely related to their functions in processes such as metastasis, angiogenesis, and immune regulation (10). In addition, Ma et al. proposed seven TAM subsets based on enriched pathways and predicted functions: IFN-induced, immune-regulatory, inflammation-rich, lipid-associated, angiogenesis-promoting, resident-tissue macrophage-like, and proliferative TAMs (18). TAMs expressing different markers also show differential spatial distribution; for instance, SPP1+macrophages are enriched in hypoxic and necrotic tumor regions, where they promote tumor progression can serve as indicators of poor prognosis (44); TAMs at tumor margin areas express high levels of interleukin 4 induced 1 protein (IL-4I1), which is related to the invasiveness of colon cancer (45); and PD-L1+ macrophages also accumulate at the tumor invasion margin, forming dense cluster-like structures (46). Metabolic characteristics also differ among subtypes, with IL-4I1+macrophages exerting immunosuppressive properties through tryptophan degradation and promoting entry of regulatory T cells into tumors (47). Finally, significant enrichment of lipid metabolism and oxidative phosphorylation pathways in lipid-associated TAMs may actively suppress anti-tumor immune responses, as lipid catabolic metabolism in macrophages is related to immunosuppression and tolerance functions, whereas lipid synthesis is associated with inflammation and immunity (18).

However, despite the increase in available data resulting from scRNA-seq studies, there is still no consensus regarding the nomenclature or functional status of TAMs. Thus, the majority of studies on TAMs reprogramming continue to use the M1/M2 polarization model. In this review, we apply the concept of reprogramming to the various changes, including polarization, exhibited by macrophage phenotypes.

## Recruitment of macrophages

Here, recruitment of macrophages mainly refers to the recruitment of monocytes or macrophages into the TME and their transformation into TAMs. Various types of immune cell can recruit macrophages via cytokines. For instance, immunotherapy-induced intracellular CD8+ T cells recruit macrophages through the C-C motif chemokine receptor 5 (CCR5) signaling axis and polarize macrophages to become M1-like TAMs, with important effects on anti-tumor immunity and immunotherapy response (48). and exhausted CD8^+^ T cells recruit monocytes to the TME and shape their differentiation to TAMs. In turn, TAMs contribute to the exhaustion of CD8^+^ T cells through antigen-specific stable synapses, in conjunction with the hypoxic environment of the tumor (49). B cells also recruit macrophages. In hepatocellular carcinoma (HCC), augmented infiltration of IgG+ plasma cells and macrophages is an indicator of poor prognosis; this is because IgG+ plasma cells are recruited by TAMs via the C-X-C motif chemokine receptor 3 (CXCR3)-C-X-C motif chemokine ligand 10 (CXCL10) axis, and IgG in turn facilitates infiltration of TAMs and expression of PD-L1 in macrophages within the tumor (50).

Myeloid-derived immune cells such as neutrophils can transform into tumor-associated neutrophils (TANs) in the TME, where they support the recruitment of TAMs. In HCC, TANs recruit macrophages and regulatory T cells to promote tumor growth, progression, and resistance to sorafenib (51). In addition, scRNA-seq analysis of 124 liver cancer patients showed that CCL4+ TANs recruited macrophages through CCL4/CCR5 signaling, and depletion of neutrophils attenuated macrophage recruitment and suppressed T cell activity, thereby inhibiting tumor growth (52).

As innate immune cells, NK cells produce interferon-γ (IFN-γ), which is essential for accumulation of monocyte-derived CD169 (also known as sialic acid binding lg like lectin 1(Siglec-1)) +TAMs in GBM. Furthermore, infiltrating CD169+ macrophages promote anti-tumor immune responses (53).

As well as immune cells, stromal cells in the TME have important roles in the recruitment of TAMs; in particular, fibroblasts transform into CAFs. In CRC, melanoma cell adhesion molecule (MCAM) in CAFs interacts with interleukin-1 receptor 1 (IL1R1) to enhance activity of the nuclear factor kappa B (NF-κB)/IL-34/CCL8 signaling pathway, thereby promoting chemotaxis of TAMs. Knockout of *MCAM* in mice has been shown to improve survival by inhibiting orthotopically injected colorectal tumor growth through reduced recruitment of TAMs (54). and mouse gastric cancer cells show enhanced expression of CXCL12 in CAFs, which promotes infiltration of TAMs. Moreover, tumor treatment with tranilast inhibits infiltration of TAMs by suppressing secretion of CXCL12, significantly promoting infiltration of CD8+ lymphocytes into the tumor and leading to apoptosis of cancer cells through an immune response (55). In addition, in CRC, increased expression of myosin regulatory light chain 9 (MYL9) in CAFs facilitates secretion of CCL2 and TGF-β1, which are linked to recruitment and infiltration of TAMs, resulting in the establishment of an immunosuppressive microenvironment that renders the tumor unresponsive to immunotherapy (56). Thus, it is clear that as well as facilitating the recruitment of macrophages, CAFs also inhibit some related pathways. Both *in vitro* and *in vivo* studies have demonstrated that estrogen receptor α (ERα)+ CAFs exert a suppressive effect on macrophage migration in prostate cancer (PCa) by inhibiting CCL5, as well as reducing IL-6 secretion, which attenuates invasiveness. Therefore, targeting signaling pathways involving CCL5 and IL-6 could represent an alternative approach to treatment of PCa (57).

The impact of CAFs on TAMs is not limited to recruitment but also includes reprogramming of macrophages. In mouse and human pancreatic cancers, CAFs and pericytes secrete elevated levels of IL-33, which leads to recruitment of TAMs and M2 polarization. Moreover, IL-33 stimulates TAMs to produce high levels of MMP9 through activation of NF-κB, and MMP9 allows tumor cells to intravasate into the circulation (58). In pancreatic ductal adenocarcinoma (PDAC), hypoxic CAFs stimulate migration and M2 polarization of macrophages in a HIF2-dependent fashion, and HIF2 inhibitor PT2399 has been found to improve response to immunotherapy in PDAC patients (59). In triple-negative breast cancer (TNBC), CAFs recruit monocytes to the tumor site through the CXCL12/CXCR4 axis and facilitate the progression of monocytes to immunosuppressive stabilin 1 (STAB1)+ lipid-associated macrophages, which are related to resistance to PD-1 blockade (60). In lung cancer, desmin-positive CAFs recruit TAMs by secreting IL-8 and promote M2 polarization of TAMs, thereby changing the composition of the TME and promoting the progression of lung cancer. Administration of IL-8 receptor antagonist SB225002 or navarixin has been demonstrated to inhibit TAMs infiltration and enhance the efficacy of anti-PD-1 or anti-PD-L1 therapy (61). Moreover, two large independent cohort studies have found the presence of CAFs with high expression of nicotinamide adenine dinucleotide (NAD) metabolic enzyme nicotinamide N-methyltransferase (NNMT) to be associated with poor prognosis in urothelial bladder cancer (UBC). These NNMT+ CAFs promote recruitment and differentiation of TAMs via serum amyloid A (SAA), leading to enhanced tumor cell proliferation and acquisition of immunotherapy resistance. Inhibition of NNMT using 5-amino-1-methylquinolinium iodide significantly suppressed tumor growth and synergistically increased the apoptotic effects of anti-PD-L1 antibody treatment (62). In addition, CAFs derived from human CRC have been shown to enhance recruitment of monocytes by upregulating vascular cell adhesion molecule 1 (VCAM-1) expression in CRC cells. CAFs attract monocytes through the IL-8-CXCR2 pathway and facilitate polarization of TAMs toward an M2 phenotype. TAMs and CAFs also synergistically suppress the function of NK cells, thereby establishing a tumor immunosuppressive microenvironment (63).

Vascular cells, namely endothelial cells and pericytes, also promote the recruitment of TAMs. Upregulation of CXCR4 in radioresistant colon cancer endothelial cells is strongly associated with recruitment of stromal cell-derived factor 1 (SDF-1, also known as CXCL12)+TAMs and their polarization toward an M2 phenotype; this can be reversed by administration of the CXCR4 antagonist AMD3100 (64). In the TME of breast cancer, EVs released by endothelial cells transfer miR-142-5p, miR-183-5p, and miR-222-3p to recruit macrophages and polarize them toward the M2-like phenotype, thereby promoting tumor growth (65).

In a xenograft mouse model of squamous carcinomas, platelet-derived growth factor subunit B (PDGF-BB)-stimulated pericytes have been found to express increased levels of IL-33, which facilitates tumor metastasis by recruiting TAMs through an IL-33-stimulation expressed gene 2 (ST2)-dependent pathway. Deletion of the gene encoding IL-33 or inhibition using a soluble ST2 receptor significantly impeded the recruitment of TAMs and inhibited metastasis (66). The fibroblast growth factor 2 (FGF2) derived from nasopharyngeal carcinoma cells (NPC) was shown to induce pericyte-specific expression of CXCL14, which in turn promoted recruitment of TAMs and their polarization to an M2-like phenotype; and lung metastasis induced by FGF2 was reduced following inhibition of TAMs using clodronate liposomes (67).

The non-cellular components of the TME, mainly the ECM, also make important contributions to the structure and function of the microenvironment. Tumors with greater and lesser degrees of matrix stiffness exhibit similar compositions with respect to cancer and stromal cell subsets. However, the presence of a stiff ECM has been shown to enhance expression of CSF1 in breast cancer cells, facilitating the recruitment of tumor-promoting M2-like macrophages (68). In addition to the physical properties of ECM, various biochemical components significantly influence the recruitment of macrophages. Hyaluronic acid (HA) in the ECM can act as a microenvironmental signal for recruitment of TAMs, and its deficiency can lead to impaired recruitment (69). HA potentially promotes macrophage recruitment and M2 polarization through the IL-1/chitinase-3-like protein 1 (CHI3L1) and TGF-β/CHI3L1 axes (70). In addition, fibronectin enhances the expression of integrin αv and α5 in TAMs and promotes M2 polarization (71). In the microenvironment of HCC, MMP21 enhances macrophage recruitment through CCL14 and promotes M2 polarization of macrophages by increasing expression levels of CSF1, ultimately facilitating tumor metastasis (72).

EVs derived from M1-polarized macrophages bind to hyaluronic acid and beta-blockers and enhance the anti-tumor activity of doxorubicin by downregulating TAMs in breast cancer (73). Prostaglandin E2 (PGE2) promotes the infiltration of M2-type macrophages in non-small cell lung cancer (NSCLC) tissues through PGE2 receptor 4 subtype (EP4); the EP4 inhibitor E7046 has been shown to block this effect and reduce tumor growth rates and vascular density (74).

**Figure 1:**
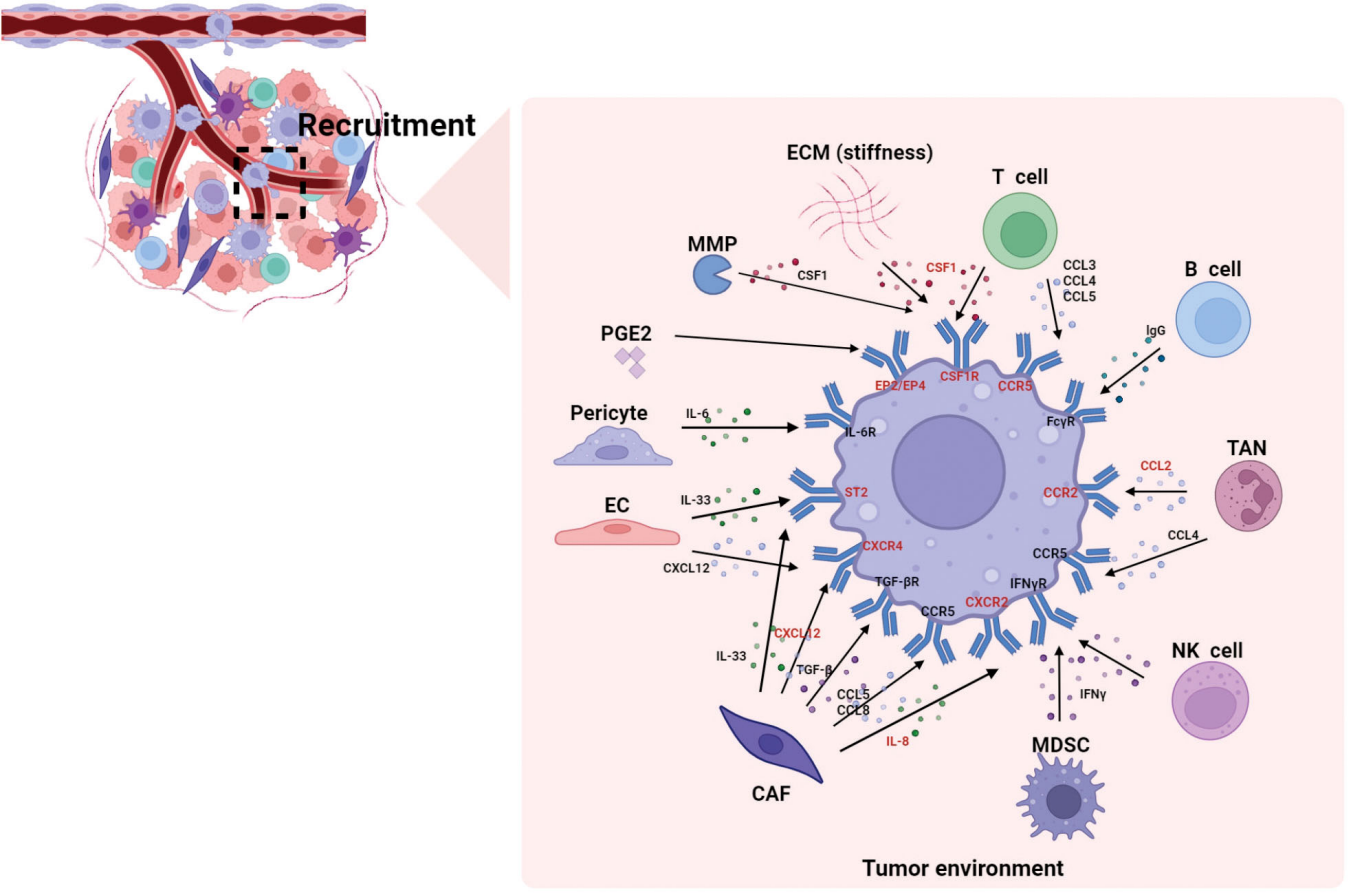
The recruitment of macrophages by different components in the TME. This figure summarizes how various components in the TME recruit macrophages. Studies indicate that chemokines and cytokines play pivotal roles in macrophages recruitment. The molecules highlighted in red represent reported macrophage-targeting molecules (e.g., CCR2, CSF1R), which hold significant research value in tumor immunotherapy. CAF, cancer-associated fibroblasts; CCL, C-C motif chemokine ligand; CCR, C-C motif chemokine receptor; CXCL, C-X-C motif chemokine ligand, CXCR, C-X-C motif chemokine receptor; CSF1R, colony stimulating factor 1 receptor; EC, endothelial cell; ECM, extracellular matrix; EP, e-type prostanoid receptor; IFN-γ, interferon-γ; IL, interleukin; MMP, matrix metalloproteinase; MDSC, myeloid-derived suppressor cell; PGE2, prostaglandin E2; ST2, stimulation expressed gene 2; TAN, tumor-associated neutrophil; TGF-β, transforming growth factor-β.

**Figure 1** summarizes how various components in the TME recruit macrophages. Undoubtedly, the chemokines and cytokines play pivotal roles in macrophages recruitment. In summary, the TME recruits monocytes and macrophages through the CSF1-CSF1R, CCL2-CCR2, CCL4-CCR5, IL-33-ST2, CXCL12-CXCR4, IL-8-CXCR2 and PGE2-EP4/EP2 pathways; thus, these pathways are major targets for potential interventions in tumor treatment. Targeting TAMs recruitment with small molecules or antibodies has been proven to be a promising therapeutic approach, either as a standalone therapy or in combination with conventional therapies.

## Reprogramming of TAMs

Previous studies have shown that reprogramming of TAMs is regulated by multiple microenvironmental cytokines, growth factors, epigenetic regulators, and other signals derived from the TME. Here, we discuss the influence of each of these components of the TME on the reprogramming of TAMs.

Regarding adaptive immune cells, CD4^+^ T cells have been reported to induce tumor-infiltrating macrophages to adopt an M1-like tumoricidal phenotype via IFN-γ, and promote the synthesis of nitric oxide (NO) in M1 through inducible nitric oxide synthetase (iNOS), resulting in indirect killing of multiple myeloma (MM) cells (75). In PDAC, CD4+ T cells were found to promote differentiation of monocytes into MHC class II anti-tumor TAMs through cognate antigen recognition and downstream CD40 and IFN-γ pathways; in turn, these MHC class II TAMs are critical promoters of anti-tumor Th1 cells and anti-tumor immunity (76). In addition, gamma-aminobutyric acid (GABA) secreted by B cells can facilitate the differentiation of monocytes into anti-inflammatory TAMs, which secrete IL-10 and impede CD8 T cell cytotoxicity, and B cell deficiency or targeted inhibition of GABA-producing enzyme augmented anti-tumor responses in a murine model of colon cancer (77).

Generally, myeloid-derived suppressor cells (MDSCs) amplify the immune-suppressive activity of macrophages and dendritic cells via crosstalk in the TME and thus limit the efficacy of cancer immunotherapies (78). For instance, MDSCs induce reprogramming of TAMs by suppressing CD40/IL-27 signals to promote melanoma progression in systemic lupus erythematosus mice. They have also been implicated in macrophage infiltration and resistance to immune checkpoint blockade (ICB) immunotherapy. Thus, preventing the reprogramming of macrophages induced by inhibition of CD40/IL-27 signals shows potential as a precise immunotherapeutic strategy in melanoma (79).As specific components of myeloid origin, platelets can also affect macrophage polarization. In CRC, augmentation of platelets within a tumor results in activation of c-Jun N-terminal kinases (JNK)/signal transducer and activator of transcription (STAT1) signaling, via binding of p-selectin to p-selectin glycoprotein ligand-1 (PSGL-1) expressed by TAMs; this facilitates activation of the complement component 5a (C5a)/C5a receptor 1 (C5aR1) axis in TAMs and causes their transformation to a tumor phenotype, thereby promoting tumor growth and metastasis. Correspondingly, inhibition of the C5a/C5aR1 axis or PSGL-1 significantly reduces the growth of CRC (80).

The impact of stromal cells such as CAFs on TAMs in the TME has also been a focus of research attention. For instance, in PDAC, CAFs secrete sialic acid, which drives the differentiation of monocytes to immunosuppressive TAMs; these TAMs were shown to have enhanced expression of PD-L1 and IL-10, and administration of a sialyltransferase inhibitor (SI) inhibited the differentiation process (81). In addition, a single-cell study of gastric cancer identified a population of CAFs expressing carnitine palmitoyltransferase 1C (CPT1C) that secreted IL-6 to facilitate the development of M2-like TAMs, thereby promoting the formation of an immunosuppressive TME (82). In recurrent osteosarcoma, significant infiltration of CAFs has been reported; moreover, these CAFs exhibited high expression of lysyl oxidase (LOX), which could induce TAM polarization and thus reshape the tumor immune microenvironment. Moreover, LOX inhibitor β-aminopropionitrile (BAPN) could effectively suppress osteosarcoma migration and promote apoptosis, and targeting LOX in CAFs in this way has demonstrated promising efficacy in treatment of recurrent osteosarcoma (83). There are various other examples. CAFs in the breast cancer TME induce polarization of macrophages toward an immunosuppressive phenotype by secreting PGE2 and TGF-β; in turn, these macrophages enhance the activation of T regulatory cells, which contribute to the establishment of an immunosuppressive microenvironment (84). In HCC, CAFs promote M2 polarization of TAMs through CXCL12 and induce secretion of plasminogen activator inhibitor 1 (PAI-1), thereby augmenting the malignant behavior of tumor cells (85). In high-grade ovarian carcinoma (HGSC), prostaglandin I2 (PGI2) synthesis is upregulated in CAFs, leading to release of PGI2 into the TME. This released PGI2 binds to the PGI2 receptor (PTGIR) on ascitic TAMs, promoting their polarization toward an immunosuppressive and pro-tumor phenotype characterized by reduced phagocytic capacity; furthermore, secretion of immune-stimulated cytokines is diminished (86). In PCa, exosomes derived from CAFs facilitate M2 polarization of TAMs through the delivery of miR-320a, thereby promoting the aggressive behavior of tumor cells (87). The melanoma-derived AKT1-carrying melanosome is released and transmitted to TAMs via dermal fibroblasts, inducing VEGF secretion in an mammalian target of rapamycin (mTOR)-dependent manner and polarization of macrophages toward a pro-tumor phenotype; this phenotypic shift has been associated with resistance to immunotherapy in melanoma (88). Integration analysis of two bladder cancer scRNA-seq datasets suggested a positive correlation between lysyl oxidase like 2 (LOXL2) expression in CAFs and expression of CD206, a marker of M2-type macrophage polarization. Furthermore, LOXL2 was identified as a potential prognostic biomarker and predictor of response to immunotherapy in bladder cancer (89). Most studies have found that CAFs facilitate the reprogramming of TAMs; however, a minority have reported contrary results. For instance, in lung adenoma cancer, secretion of stanniocalcin-1 (STC1) by CAFs was found to inhibit differentiation and maturation of TAMs through sequestration of glucose regulated protein 94 (GRP94) an autocrine macrophage-differentiation-inducing factor, by binding to its cognate scavenger receptor, thereby hindering the development of cancer (90). These findings amply demonstrate the complexity of the microenvironment and the diversity of TAMs.

Other stromal cells also affect the reprogramming of TAMs. For instance, endothelial-to-mesenchymal transition (EndoMT) can provide a source of CAFs; and the heat shock protein 90 alpha (HSP90α) secreted by endogenous EndoMT-derived cells can induce polarization of macrophages toward M2 and promote secretion of more HSP90α to facilitate the growth of PDAC tumors. Furthermore, in animal models, an anti-HSP90α antibody has demonstrated remarkable therapeutic efficacy against PDAC tumors (91). Moreover, osteopontin secreted by myofibroblastic metastasis-associated fibroblasts (myMAFs) promotes an immunosuppressive macrophage phenotype, whereas pharmacological blockade of STAT3 or myMAF-specific genetic depletion of STAT3 restores an anti-tumor immune response and reduces PDAC liver metastasis (17). The presence of myeloid differentiation primary response protein 88 (MyD88) in myofibroblasts can augment M2 polarization of macrophages via secretion of CCL9, thereby facilitating the development of HCC associated with non-alcoholic fatty liver disease. This process may rely on activation of the CCR1 receptor; indeed, administration of CCR1 inhibitors has been shown to inhibit tumor growth in mice, suggesting potential molecular targets for HCC therapy (92). In addition, cardiac mesenchymal stromal cell-derived EVs (cMSC-SeV) can activate macrophages to promote angiogenesis and tumor generation after myocardial infarction, thereby facilitating progression and metastasis of lung cancer. This is a possible explanation for the elevated incidence of tumors in patients with myocardial infarction (93).

The TME also contains vascular cells, which encompass endothelial cells and pericytes and are also associated with reprogramming of TAMs. For instance, direct interactions with endothelial cells and responses to CSF1 signals secreted by these cells are crucial for the formation and maintenance of macrophage colonies. Endothelial cells can also facilitate the polarization of macrophages toward an M2 phenotype that exhibits high expression of VEGFα and have been shown to promote angiogenesis and tumor progression in this way in mouse models of PCa (94). In GBM, tumor-associated endothelial cells induce HIF-2α-dependent upregulation of Arg1 expression through secretion of IL-6, thereby promoting M2 polarization of TAMs and facilitating cancer progression. Conversely, specific knockdown of IL-6 in endothelial cells inhibits alternative activation of TAMs and has been shown to enhance survival rates of GBM-bearing mice (95).

Finally, the non-cellular ECM component of the TME is also involved in reprogramming of TAMs. The ECM has significant roles in cytoskeleton support, biomechanical transduction, and biochemical signal transformation, and ECM-educated macrophages have been found to share transcriptional profiles with TAMs from ovarian metastasis tissue (96). Moreover, in solid tumors, the functionality of TAMs is modulated by the stiffness of the ECM; mechanical signals induced by ECM stiffness can activate mechanoreceptors on the cell membrane and the corresponding mechanotransducers in the cytoplasm, thereby regulating the phenotypes and polarization of TAMs (97). High collagen density also promotes the acquisition immunosuppressive phenotypes by macrophages; this explains the observed association between high collagen density and poor prognosis (98). In addition, as discussed above, the metabolic reprogramming of macrophages affects the metabolic products of the ECM, and these metabolic products in turn influence the functions of macrophages. For instance, lactate, a product of glycolysis, induces the differentiation of TAMs into the M2 phenotype, which is characterized by increased expression of VEGF (39).

**Figure 2:**
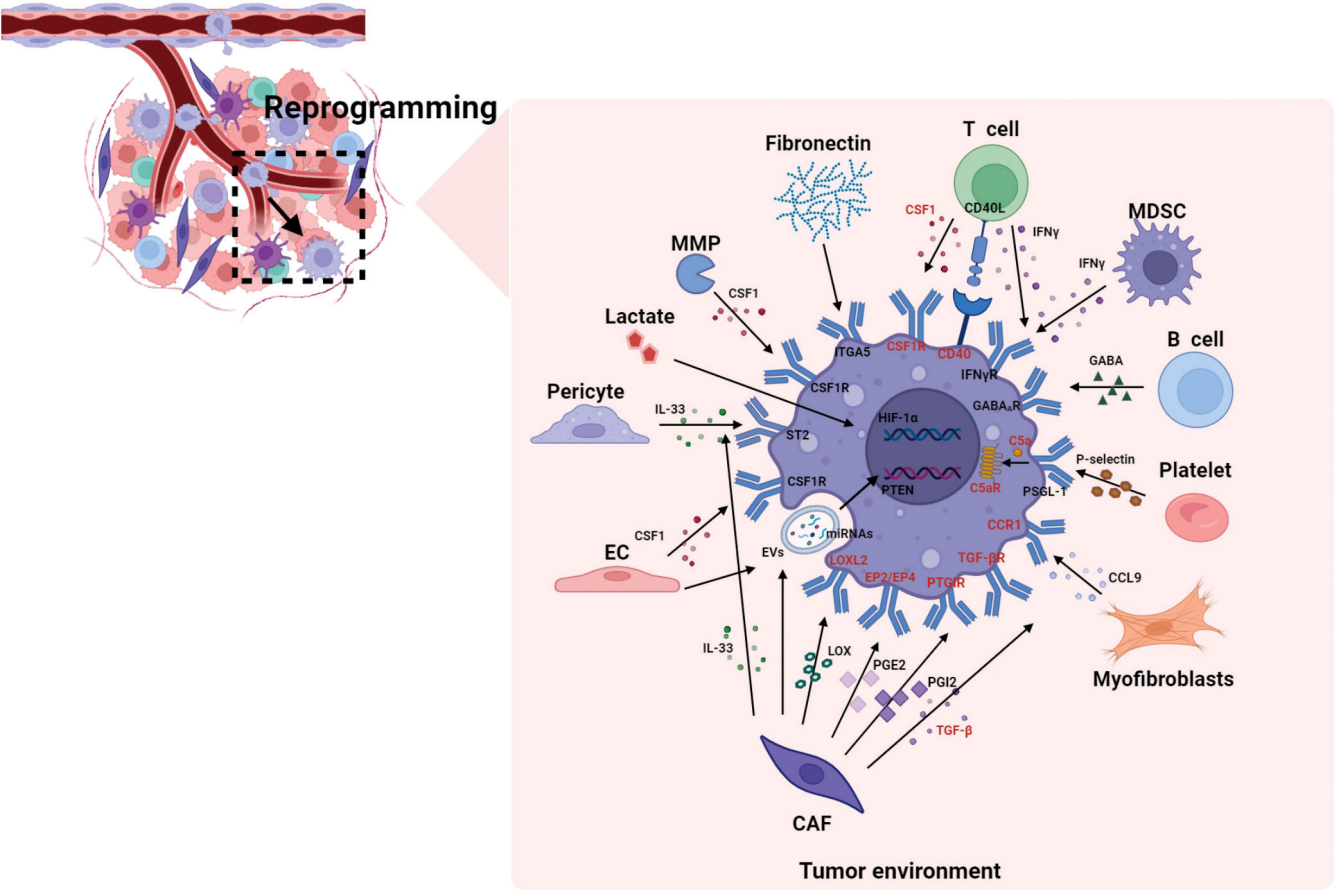
The reprogramming of macrophages by different components in the TME. This figure summarizes how various components in the TME affect the reprogramming of macrophages. The cellular components in the TME regulate reprogramming through cytokines and chemokines, as well as through soluble proteins, metabolites (such as lactate), EVs, etc. And the reprogramming process involves not only epigenetic regulation of critical transcription factors (such as HIF-1α), but also the activation of complement cascades (C5a/C5aR signaling axis), Similarly, the highlighted molecules refer to the reported targets for macrophages. C5aR, complement component 5a receptor; CAF, cancer-associated fibroblasts; CCL, C-C motif chemokine ligand; CCR, C-C motif chemokine receptor; CSF1R, colony stimulating factor 1 receptor; EC, endothelial cell; ECM, extracellular matrix; EP, e-type prostanoid receptor; EVs, extracellular vesicles; GABA, gamma-aminobutyric acid; HIF-1α, hypoxia inducible factor 1α; IFN-γ, interferon-γ; IL, interleukin; ITGA5, integrin α5; LOXL2, lysyl oxidase like 2; MDSC, myeloid-derived suppressor cell; MMP, matrix metalloproteinase; miRNAs, microRNAs; PGE2, prostaglandin E2; PGI2, prostaglandin I2; PTGIR, prostaglandin I2 receptor; PSGL-1, p-selectin glycoprotein ligand-1; PTEN, phosphatase and tensin homolog; ST2, stimulation expressed gene 2; TAN, tumor-associated neutrophil; TGF-β, transforming growth factor-β.

**Figure 2** summarizes how various components in the TME affect the reprogramming of macrophages. The reprogramming of macrophages involves not only cytokines and chemokines, but also soluble proteins, metabolites, EVs, etc. Moreover, TAMs possess distinct activation states and subpopulations and demonstrate significant functional plasticity. This enables them to rapidly adapt to changes in the TME and precisely coordinate their functions in response to these changes (8, 23, 99), The dynamic interactions between TAMs and the TME can vary based on the specific activation states of TAMs. Therefore, investigation of all the components of the TME that influence reprogramming of TAMs, as well as the mechanisms by which they exert their influence, is essential for developing novel therapeutic strategies to reverse macrophage polarization and inhibit tumor growth.

## Functional modulation of TAMs

The primary functions of macrophages encompass phagocytosis, secretion of cytokines, and antigen presentation. Functional modulation refers to the modification of specific functions of TAMs without alteration of their overall phenotype. A typical example of this process involves CD47, a checkpoint on macrophages. Blockage of the CD47-signal regulatory protein α (SIRPα) signaling pathway can enhance the phagocytic function of macrophages toward tumor cells, but this does not significantly alter the polarization state of the macrophages (100). Regarding functional regulation of TAMs more generally, studies of the regulatory effects of tumor cells have been more prevalent; there has been relatively little research on the effects of the microenvironment.

In the bone marrow microenvironment of MM, malignant plasma cells release mitochondrial DNA to activate macrophages and promote chemokine-induced upregulation of macrophages via stimulator of interferon genes (STING) signaling; these mtDNA-activated TAMs promote MM progression and retention of MM cells in the pro-tumoral bone marrow microenvironment. Moreover, STING inhibition (with H-151) reduces MM tumor burden (101). In a mouse model of colon adenocarcinoma with a Th1-dominant TME, polarization toward Th1 cells enhanced the immunosuppressive activity of TAMs in the presence of elevated levels of IFN-γ (102). In HCC, CXCL10 produced by macrophages binds to CXCR3 on B cells, inducing their differentiation into IgG-producing plasma cells. The secretion of IgG by plasma cells can activate Fc gamma receptors (FcγR) on macrophages, leading to production of IL-6, IL-10, and CCL20 while suppressing the anti-tumor immune response. Depletion of B cells and antibody blockade of FcγR has been shown to prevent generation of these macrophages, as well as enhancing anti-tumor T cell responses and inhibiting growth of liver cancer (103).

Regarding the ECM, high collagen density has been shown to induce macrophages to acquire immunosuppressive phenotypes; these macrophages inhibit T cell proliferation and have decreased ability to attract CD8+ T cells (98). In addition, adenosine increases the expression of molecular markers of alternative-type (M2) macrophages, including Arg1 and tissue inhibitor of MMP1 (104). As PCa progresses, a subpopulation of TAMs with high expression of SPP1 accumulates, resulting in inhibition of the activity of CD8+ T cells through the adenosine signaling pathway. However, blocking the adenosine A2A receptor (A2AR) effectively reduces the immunosuppressive effects of these TAMs (105).

The microenvironment can also exert inhibitory effects on TAMs. Appropriately stimulated NK cells isolated from ascites of ovarian cancer patients have been shown to efficiently kill TAMs expressing low levels of HLA class I molecules, thereby reducing numbers of macrophages with tumor-promoting properties (106).

**Table 1** summarizes the effects of cellular and non-cellular components of the TME on macrophages. The influence of the microenvironment on macrophage function is frequently intertwined with reprogramming. Moreover, recruitment and reprogramming often occur concurrently, immune cells tend to have stronger effects on TAM recruitment, whereas stromal cells exert a greater influence over TAM reprogramming.

**Table 1:** Impact of various components of the tumor microenvironment on macrophages.

Immune cells							
Different components	Impact on macrophages	Tumor model	Molecules in the TME		Targets on TAMs	Inhibitors	Ref.
CD8+ T cells	1.Recruitment 2.Reprogramming	Melanoma	CCL3/CCL4/CCL5		CCR5	Maraviroc (CCR5 inhibitor)	(48)
Exhausted CD8+T cells	1.Recruitment 2.Reprogramming	Melanoma	CSF1		CSF1R	Anti-CSF1R antibody	(49)
CD4+ T cells	Reprogramming	MM	IFN-γ		iNOS	NA	(75)
CD4+ T cells	Reprogramming	PDAC	IFN-γ/CD40L		CD40	NA	(76)
Th1 cells	Functional modulation	Colon cancer	IFN-γ		NA	NA	(102)
B cells	Recruitment	HCC	IgG		NA	NA	(50)
B cells	Reprogramming	CRC	GABA		NA	NA	(77)
Plasma cells	Functional modulation	MM	mtDNA		STING	H-151 (STING inhibitor)	(101)
Plasma cells	Functional modulation	HCC	lgG		FcγR	FcγR blocking	(103)
TANs	Recruitment	HCC	CCL2		CCR2	Anti-CCL2 antibody	(51)
TANs	Recruitment	Liver cancer	CCL4		CCR5	NA	(52)
MDSCs	1.Recruitment 2.Reprogramming	Melanoma	IFN-γ		CD40/IL-27	Agonist CD40 antibody	(79)
Platelets	Reprogramming	CRC	P-selectin		PSGL-1/C5a-C5aR1	Anti-C5aR1 antibody	(80)
NK cells	Recruitment	GBM	IFN-γ		CD169	NA	(53)
NK cells	Functional modulation	Ovarian cancer	NA		MHC-I	NA	(106)
Stromal cells							
CAFs	Recruitment	CRC	MCAM-IL1R1-NF-ĸB-IL34/CCL8		NA	NA	(54)
CAFs	Recruitment	GC	CXCL12		CXCR4	Tranilast	(55)
CAFs	Recruitment	CRC	MYL9-CCL2/TGF-β1		NA	NA	(56)
CAFs	Recruitment	PCa	ERα-CCL5/IL6		NA	NA	(57)
CAFs	1.Recruitment 2.Reprogramming	PDAC	IL-33		ST2-NF-ĸB-KMMP9	A soluble ST2 receptor	(58)
CAFs	1.Recruitment 2.Reprogramming	PDAC	HIF-2α		NA	PT2399 (HIF2 inhibitor)	(59)
CAFs	1.Recruitment 2.Reprogramming	TNBC	CXCL12		CXCR4	NA	(60)
CAFs	1.Recruitment 2.Reprogramming	Lung cancer	IL-8		IL-8R/CXCR2	SB225002 or navarixin (IL-8R antagonist)	(61)
CAFs	1.Recruitment 2.Reprogramming	UBC	NNMT-SAA3		NF-ĸB	5-amino-1-methylquinolinium iodide (NNMT Inhibitor)	(62)
CAFs	1.Recruitment 2.Reprogramming	CRC	IL-8		CXCR2	Danirixin (CXCR2 antagonis) /Anti-IL-8 antibody	(63)
CAFs	Reprogramming	PDAC	Sialic acid		Siglec-9	Sialyltransferase inhibitor (SI)	(81)
CAFs	Reprogramming	GC	IL-6		NA	NA	(82)
CAFs	Reprogramming	OS	LOX		NA	BAPN (LOX inhibitor)	(83)
CAFs	Reprogramming	Mammary gland tumor	TGF-β/ PGE2		EP2/EP4	SB505125 (TGF-β receptor inhibitor)/ L161982 (EP2 inhibitor)/ Celecoxib	(84)
CAFs	Reprogramming	HCC	CXCL12		PAI-1	NA	(85)
CAFs	Reprogramming	HGSC	PGI2		PTGIR	CAY10449 (PTGIR antagonist)	(86)
Exosomesfrom CAFs	Reprogramming	PCa	miRNA-320a		PTEN/PI3Kγ	NA	(87)
Melanosomes from CAFs	Reprogramming	Melanoma	AKT1		mTOR-VEGF	Capivasertib (AKT inhibitor)	(88)
CAFs	Reprogramming	Lung cancer	STC1		GRP94	NA	(90)
EndoMT cells	Reprogramming	PDAC	HSP90α		NA	Anti-HSP90α antibody	(91)
MyMAFs	Reprogramming	PDAC	STAT3-osteopontin		NA	Silibinin (STAT3 inhibitor)	(17)
Myofibroblasts	Reprogramming	HCC	CCL9		CCR1	J113863 (CCR1 inhibitor)	(92)
cMSC-SeV	Reprogramming	Lung cancer	Tumor-promoting cytokines, proteins, and miRNAs		NA	NA	(93)
Vascular cells							
Endothelial cells	1.Recruitment 2.Reprogramming	Colon cancer	CXCR4	CXCL12 (SDF-1)		AMD3100 (CXCR4 antagonist)	(64)
Endothelial EVs	1.Recruitment 2.Reprogramming	Breast cancer	miR-142-5p/miR-183-5p/miR-222-3p	PTEN		NA	(65)
Endothelial cells	Reprogramming	PCa	CSF1	CSF1R		GW2580 (CSF1R inhibitor)	(94)
Endothelial cells	Reprogramming	GBM	IL-6	HIF-2α		NA	(95)
Pericytes	Recruitment	Squamous carcinoma	IL-33	ST2		A soluble ST2 receptor	(66)
Pericytes	1.Recruitment 2.Reprogramming	PDAC	IL-33	ST2-MMP9		A soluble ST2 receptor	(58)
Pericytes	1.Recruitment 2.Reprogramming	NPC	CXCL14	NA		Clodronate liposome	(67)
ECM							
Stiffness	Recruitment	Breast cancer	CSF1	CSF1R		CSF1R inhibitor	(68)
HA	1.Recruitment 2.Reprogramming	Gliomas	TGF-β-CHI3L1	NA		NA	(70)
MMP21	1.Recruitment 2.Reprogramming	HCC	CCL14 and CSF1	CSF1R		NA	(72)
Fibronectin	Reprogramming	Breast tumor	Fibronectin	integrins αv and α5		NA	(71)
Metabolites							
PGE2	Recruitment	NSCLC	PGE2	EP4		E7046 (EP4 inhibitor)	(74)
Lactate	Reprogramming	Melanoma /Colon carcinoma	Lactate	HIF-1α		NA	(39)
Adenosine	Functional modulation	PCa	Adenosine	A2AR		Ciforadenant (A2AR inhibitor)	(105)

A2AR, adenosine A2A receptor; BAPN, β-aminopropionitrile; C5a, complement component 5a; C5aR1, C5a receptor 1; CCL8, C-C motif chemokine ligand 8; cMSC-SeV, cardiac mesenchymal stromal cell-derived extracellular vesicles; CAFs, cancer-associated fibroblasts; CHI3L1, chitinase-3-like protein 1; CRC, colorectal cancer; CSF, colony stimulating factor 1; ECM, extracellular matrix; EndoMT, endothelial-to-mesenchymal transition; EP2/EP4, prostaglandin E2 receptor 2/4; GABA, gamma-aminobutyric acid; GBM, glioblastoma; GC, gastric cancer; GRP94, glucose regulated protein 94; HA, hyaluronic acid; HCC, hepatocellular carcinoma; HGSC, high-grade ovarian carcinoma; HIF2, hypoxia inducible factor 2; IL1R1, interleukin-1 receptor 1; IL34, interleukin-34; iNOS, inducible nitric oxide synthetase; LOX, lysyl oxidase; MCAM, melanoma cell adhesion molecule; MM, multiple myeloma; mtDNA, mitochondrial mTOR, mammalian target of rapamycin; MYL9, myosin regulatory light chain 9; DNA; MyMAFs, myofibroblastic metastasis-associated fibroblasts; NA, not applicable; NF-ĸB, nuclear factor kappa B; NNMT, nicotinamide N-methyltransferase; NPC, nasopharyngeal carcinoma; NSCLC, non-small cell lung cancer; OS, osteosarcoma; PAI-1, plasminogen activator inhibitor-1; PCa, prostate cancer; PDAC, pancreatic ductal adenocarcinoma; PGE2, prostaglandin E2; PI3Kγ, phosphatidylin-ositol-3-kinase gamma; PSGL-1, p-selectin glycoprotein ligand-1; PTEN, phosphatase and tensin homolog; SAA, serum amyloid A; Siglec-9, sialic acid binding lg like lectin 9; STC1, stanniocalcin-1; TGF-β, transforming growth factor β; TNBC, triple-negative breast cancer; VCAM-1, vascular cell adhesion molecule-1; VEGF, vascular endothelial growth factor; UBC, urothelial bladder cancer.

## Clinical trials targeting TAMs

Existing clinical strategies targeting TAMs involve TAM depletion, inhibition of TAM recruitment, reprogramming of TAMs, and chimeric antigen receptor macrophages (CAR-M) (107). The strategies involving inhibition of recruitment and reprogramming of macrophages are the most closely related to the influence of the TME on TAMs and thus to the points discussed in the present review. TAM depletion has achieved limited success in clinical trials to date, although a CAR-T-based strategy targeting macrophage marker F4/80 (F4.CAR-T) for TAM depletion has been tested in mouse models of solid tumors. F4.CAR-T cells infiltrated the tumor lesion, delaying tumor growth, and significantly prolonging the survival of mice with non-small cell lung carcinoma compared with those treated with PD-1 blockade.

As for TAMs depletion, it has achieved limited success in the clinical trials of the methods developed to date. Using chimeric antigen receptor T cells (CAR-T) targeting the macrophage marker F4/80 (F4.CAR-T), a strategy for TAMs depletion in mouse solid tumor models was achieved. Compared with PD-1 blockade, F4.CAR-T cells infiltrated the tumor lesion, delayed tumor growth, and significantly prolonged the survival of mice with NSCLC (108). CAR-M is considered to be a promising approach for treatment of solid tumors, because as well as transforming pro-tumor M2 macrophages into anti-tumor M1 macrophages, it enhances the antigen presentation mechanism to recruit and present antigens to T cells (109). Numerous instances have been reported in which one anti-TAM drug can affect multiple processes simultaneously; for instance, a study found that CSF1R inhibitors could inhibit recruitment, reprogramming, and function (99). CSF1R expression is restricted to macrophages at the tumor site, indicating that CSF1 may promote metastatic potential by regulating the infiltration and function of TAMs (110).

There have been many clinical trials of drugs targeting TAMs, either as monotherapies or in combination with chemotherapy and immunotherapy. For example, engineered microparticles loaded with resiquimod (R848@M2pep-MPsAFP) in macrophages overexpressing alpha-fetoprotein (AFP) have been shown to target and reprogram immunosuppressive M2-like TAMs into the M1-like phenotype, thereby enhancing anti-PD-1 therapy for HCC (111). Future advances in anti-tumor therapies targeting macrophages should consider a multifaceted approach to optimize therapeutic outcomes. Given the various distinct effects of different components of the TME on TAMs, various types of inhibitors have been identified; these are summarized in **Table 2**. Here, we focus particularly on drugs that modulate TME-TAM interactions to exert their anti-tumor effects.

**Table 2:** Selected clinical trials of agents targeting tumor-associated macrophages.

Recruitment					
Classification	Compound	Clinical Phase	Tumor Model	Combination Partners	NCT Identifier
Anti-CCL2 mAb	Carlumab (CNTO 888)	Phase II	PCa	NA	NCT00992186
Anti-CCR2 mAb	Plozalizumab (MLN1202)	Phase II	Solid tumors with bone metastases	NA	NCT01015560
CCR2 inhibitor	CCX-872	Phase Ib	PDAC	Chemotherapy (FOLFIRINOX)	NCT02345408
Anti-CCR5 mAb	Leronlimab (PRO 140)	Phase Ib/II	TNBC	Carboplatin	NCT03838367
CCR5 inhibitor	Maraviroc	Phase I	Metastatic colorectal cancer	Pembrolizumab	NCT03274804 (112)
ST2 inhibitor	NEROFE	Phase I	*KRAS*-mutated ST2-positive solid tumor	doxorubicin	NCT05661201
CXCL12 inhibitor	Olaptesed pegol (NOX-A12)	Phase I/II	GBM	Bevacizumab	NCT04121455
Anti-CXCR4 mAb	Ulocuplumab	Phase I/II	Acute myeloid leukemia	Cytarabine	NCT02305563
CXCR4 inhibitor	Balixafortide	Phase III	HER2 negative breast cancer	Eribulin	NCT03786094
Anti-IL-8 mAb	BMS-986253 (HuMax-IL8)	Phase Ib/II	PCa	Nivolumab	NCT03689699
CXCR2 inhibitor	Navarixin (MK-7123)	Phase II	Advanced solid tumors	Pembrolizumab	NCT03473925
Recruitment and Reprogramming					
Anti-CSF1R mAb	Emactuzumab	Phase II	Ovarian cancer	Paclitaxel and Bevacizumab	NCT02923739
CSF1R inhibitor	Pimicotinib (ABSK021)	phase III	Tenosynovial giant cell tumor	NA	NCT05804045
Anti-CSF1 mAb	Lacnotuzumab (MCS110)	Phase II	TNBC	Gemcitabine and Carboplatin	NCT02435680 (113)
CSF1 inhibitor	HMPL-653	Phase I	Advanced solid tumors	NA	NCT05277454
Reprogramming					
CD40 agonist (antibody)	Mitazalimab (ADC-1013)	Phase Ib/II	Pancreatic cancer	Chemotherapy (mFOLFIRINOX)	NCT04888312
CD40 agonist (fusion protein)	SL-172154 (SIRPα-Fc-CD40L)	Phase Ib	Ovarian cancer	Liposomal Doxorubicin or Mirvetuximab	NCT05483933
CD40 agonist (oncolytic virotherapy)	LOAd703	Phase I/II	PDAC	paclitaxel plus gemcitabine	NCT04123470 (114)
Anti-C5a mAb	Vilobelimab (IFX-1)	Phase ​II	Cutaneous squamous cell carcinoma	pembrolizumab	NCT04812535
Anti-C5aR mAb	Avdoralimab (IPH 5401)	Phase I	Advanced solid tumors	Durvalumab	NCT03665129
TGF-β ASOs	Trabedersen (AP12009)	Phase IIB	GBM	NA	NCT00431561
TGF-β vaccine	Lucanix	Phase III	NSCLC	NA	NCT00676507
TGF-β receptor inhibitor	Galunisertib (LY2157299)	Phase II	PCa	Enzalutamide	NCT02452008
STING Agonist	E7766	Phase I/Ib	Advanced solid tumors	NA	NCT04144140
Anti-LOXL2 mAb	Simtuzumab	Phase II	PDAC	Gemcitabine	NCT01472198
EP4 antagonist	E7046	Phase I	Advanced tumors	NA	NCT02540291 (115)
EP2/EP4 dual antagonists	OKN4395	Phase I	Solid tumors	Pembrolizumab	NCT06789172
Functional modulation					
A2AR inhibitor	Ciforadenant	Phase I/Ib	Advanced tumors	Atezolizumab	NCT02655822

A2AR, adenosine A2A receptor; ASOs, antisense oligonucleotides; C5a, complement component 5a; C5aR1, C5a receptor 1; CCL2, C-C motif chemokine ligand 2; CSF, colony stimulating factor 1; EP2/EP4, prostaglandin E2 receptor 2/4; GBM, glioblastoma; mAb, monoclonal antibody; LOXL2, NA, not applicable; NSCLC, non-small cell lung cancer; PCa, prostate cancer; PDAC, pancreatic ductal adenocarcinoma; TGF-β, transforming growth factor β; TNBC, triple negative breast cancer.

**Figure 3:**
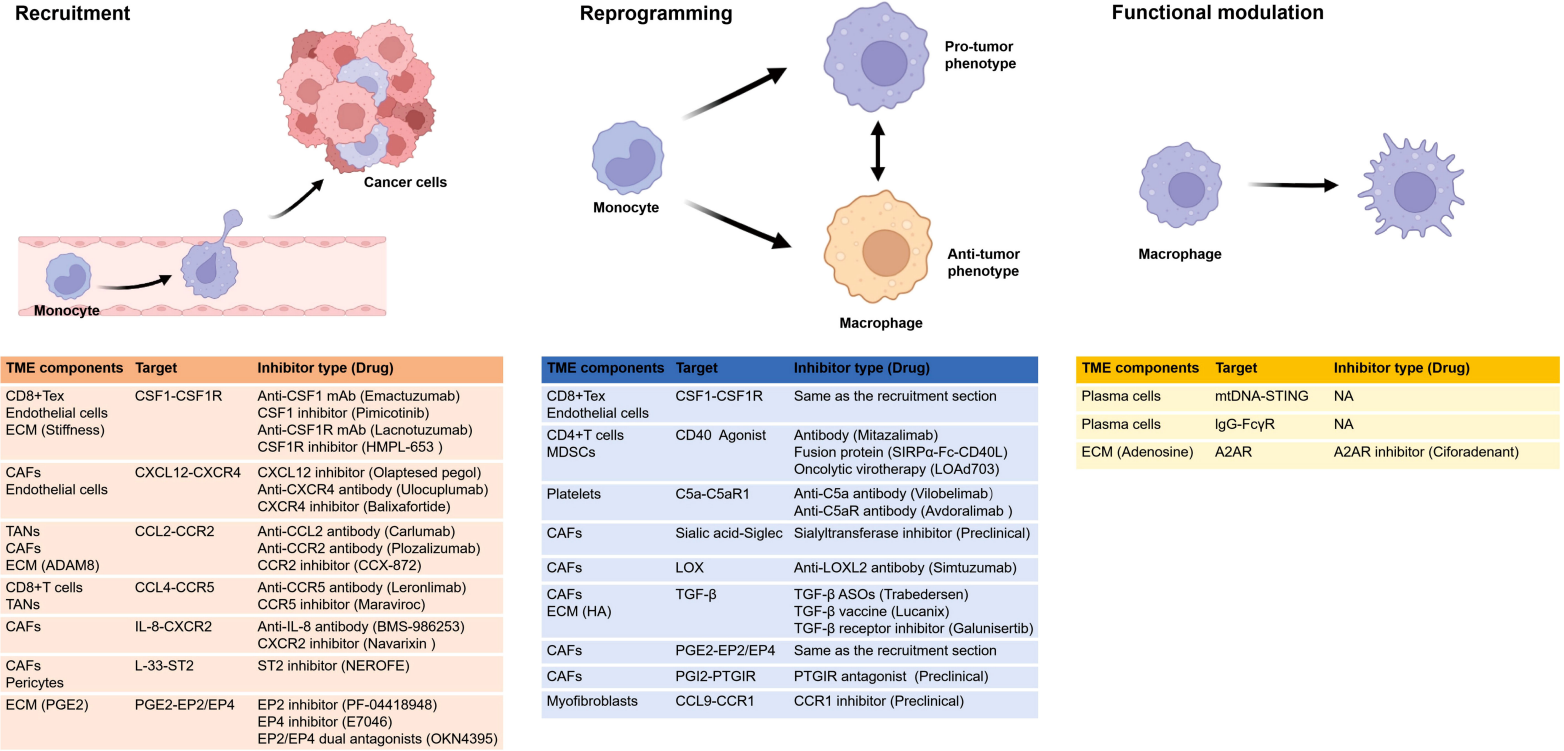
Various types of drugs from the perspective of the distinct impacts of the TME on TAMs. The figure shows the recruitment, reprogramming, and functional alterations of TAMs influenced by the TME. It enumerates how various TME components influence TAMs through distinct targets and highlights corresponding inhibitors, with one representative drug in clinical trials provided for each inhibitor. Notably, research on functional impacts remains limited. and certain drugs can affect more than one process, such as CSF1 inhibitors and EP4 inhibitors, that inhibit recruitment and reprogramming. A2AR, adenosine A2A receptor; ADAM8, a disintegrin and metalloprotease 8; CAFs, cancer-associated fibroblasts; CSF1R, colony stimulating factor 1 receptor; ECM, extracellular matrix; EP, e-type prostanoid receptor; HA, hyaluronic acid; LOX, lysyl oxidase; MDSC, myeloid-derived suppressor cells; mtDNA, mitochondrial DNA; NA, not applicable; PGE2, prostaglandin E2; PGI2, prostaglandin I2; PTGIR, prostaglandin I2 receptor; ST2, stimulation expressed gene 2; STING, stimulator of interferon genes; TANs,tumor-associated neutrophils. All figures are created in https://BioRender.com.

**Figure 3** shows various types of anti-TAM drugs from the perspective of the distinct impacts of the TME on TAMs. Therapeutic strategies that aim to modify recruitment of TAMs via targeting these cells with small molecules or antibodies have shown some promise; some key target pathways including CSF1-CSF1R, CCL2-CCR2, CCL4-CCR5, IL-33-ST2, CXCL12-CXCR4, IL-8-CXCR2, PGE2-EP2/EP4, most of which involve cytokines. Inhibiting or enhancing a single cytokine or chemokine pathway may not yield sustained anti-tumor efficacy, and monotherapies targeting these pathways have demonstrated limited clinical benefit. However, the overall safety profile of such treatments remains favorable (116), hence their current use in combination with other cancer therapies.

Regarding reprogramming, various signaling molecules can reprogram macrophages into an anti-tumor state. Based on their mechanisms of action, CD40, TLRs and STING agonists have been developed to target macrophages, with the aim of reinstating immune surveillance and reducing tumor growth. In clinical trials, these agents are frequently combined with chemoradiotherapy, other targeted therapies, or immunotherapy. CD40 has a crucial role in macrophage reprogramming by T cells and MDSCs (76, 79), and CD40 agonists include antibodies, fusion proteins, and viral vectors. STING signaling is required for plasma-cell-mediated macrophage activation and cytokine secretion. In studies of MM, STING inhibition (by H-151) has been shown to reduce tumor burden and prolong survival; this is in contrast to findings in other cancer models but reflects the specificity of different components of the TME (101). No role of TLRs in the interaction between the TME and TAMs has yet been reported.

Apart from the three pathways, we have also summarized other possible pathways or molecular-related clinical experiments that may affect reprogramming, such as CSF1-CSF1R, C5a-C5aR1, PGI2-PTGIR, CCL9-CCR1, Sialic acid-Siglec, LOX, TGF-β, PGE2-EP2/EP4. Among them, blocking the CSF1-CSF1R signaling pathway can not only reduce the recruitment and differentiation of TAMs, but also promote the reprogramming of TAMs to the anti-tumor type, thereby enhancing the anti-tumor immune response (68, 94, 99). Recent studies have identified the C5a/C5aR axis as a novel immune checkpoint, with clinical evidence suggesting that targeting this pathway may represent an effective strategy for tumor immunotherapy. Specifically, activation of the C5a/C5aR1 axis in TAMs promotes their transformation into a tumor-supportive phenotype, and inhibition of the C5a/C5aR1 axis has been shown to reduce CRC growth (80). And TGF-β recruit macrophages and promote the immunosuppressive reprogramming of TAMs (84, 117). TGF-β has critical roles in tumor progression, immune evasion, and resistance to immunotherapy within the TME (118). Various strategies have been developed to exploit the therapeutic potential of blocking TGF-β in tumor immunotherapy; these include antisense oligonucleotides (ASOs), neutralizing antibodies, engineered fusion proteins, and small-molecule inhibitors (119).

There are also drugs targeting LOX and LOXL2, however, clinical trials mainly focus on diseases related to fibrosis. Simtuzumab is a monoclonal antibody targeting LOXL2. In the Phase II clinical trial of pancreatic cancer, it was halted because it failed to improve the progression-free survival (PFS) of patients (NCT01472198) (120). The inhibitors targeting EP2/EP4 include three types: EP2 inhibitors, EP4 inhibitors and EP2/EP4 dual antagonists. And the EP2 inhibitor PF-04418948 has shown potential to enhance the therapeutic efficacy of hypopharyngeal squamous cell carcinoma (HPSCC) in preclinical studies (121). Currently, it is only undergoing Phase I clinical trials in healthy volunteers (NCT01002963), and has not yet been applied to tumor patients. E7046 is a highly selective antagonist of EP4, and in the phase I clinical trial for patients with advanced malignant tumors, E7046 demonstrated controlled tolerability and immunomodulatory effects (NCT02540291) (115). The main clinical studies on drugs targeting PGI2-PTGIR are concentrated on pulmonary fibrosis and pulmonary arterial hypertension. Meanwhile, the inhibitors of sialyltransferase have also been developed, although these are mostly still at the stage of preclinical studies.

As for functional modulation, relevant studies are relatively scarce. Among them, A2AR antagonists may have played a role. Ciforadenant is an A2AR selective antagonist. When used in combination with anti-PD-1, it may enhance the anti-tumor effect in patients with renal cancer (122). *In vitro* experiments show that ciforadenant significantly reverse the CD8+ T cell immunosuppression mediated by SPP1^hi^-TAMs and enhance the efficacy of immune checkpoint inhibitors (ICIs) (105).

**Table 2** provides a summary of selected clinical trials of drugs targeting TAM recruitment, reprogramming and functional modulation, with one representative drug listed for each category. Combination therapies are crucial for the success of anti-tumor immunotherapy, which is frequently employed in conjunction with chemoradiotherapy or T cell immunotherapy (123). Given the extensive range of potential therapeutic targets and strategies, as well as the variability in immune landscapes among patients, more rigorous preclinical studies are imperative for therapies targeting TAMs (116). Although preclinical studies to date have demonstrated that these drugs do influence TAM recruitment, reprogramming, and function, they have not yet validated the anticipated changes in TAMs within the tumor tissues of patients undergoing treatment.

## Conclusions and perspectives

Macrophages exhibit a high degree of cellular heterogeneity. Studies have confirmed that they retain their normal functionality upon reintegration into mice after prolonged cultivation in laboratory settings, without discernible differentiation from resident tissue macrophages (124). This implies that macrophages can adapt in terms of their phenotype in response to diverse microenvironments. Therefore, TAMs exhibit significant variations in morphology, biochemistry, phenotype, and function. For example, macrophage receptor with collagenous structure (MARCO)+ TAMs augment proliferation of regulatory T cells and production of IL-10 while suppressing activity of CD8+ T cells (14), whereas nucleotide binding oligomerization domain containing 1 (NOD1)+ TAMs activate CD8+ T cells (125). However, the heterogeneity and diversity of TAMs pose a formidable challenge when translating preclinical research findings into clinical practice.

Treatment strategies based on macrophages can complement and synergize with traditional tumor therapies (126). For instance, CAR-M has emerged as a highly promising anti-tumor therapeutic candidate, with continuous development and optimization since Biglari et al. first engineered carcinoembryonic antigen (CEA)-targeting CAR molecules in human monocytes in 2006. Currently, most CAR-M-based therapies are still at the animal experimentation stage; however, they have demonstrated favorable effects in certain diseases (127). For instance, in mice, CAR-M targeting uPAR has been shown to specifically localize to the liver and promote the recruitment of neutrophils and M2 macrophages, thereby reducing liver fibrosis and improving liver function. Importantly, no adverse effects were observed in the mice during CAR-M treatment (128). Macrophages derived from induced pluripotent stem cells (iMacs) have greater research value with respect to immune-cell-based tumor therapy compared with those derived in the traditional manner from autologous peripheral blood mononuclear cells. Genetically engineered CAR-iMacs have shown the potential to effectively modulate the TME (129). For instance, mesothelin-targeted CAR-iMacs have exhibited excellent targeted killing of ovarian cancer cells, demonstrating their promise in therapeutic applications. Introduction of CAR also significantly enhances the expression of inflammatory factors including IL-1B, IL-6, and TNF-α, indicating that CAR-iMacs can improve the immune microenvironment upon recognition of specific antigens (130). Second-generation CAR-iMacs have achieved antigen-dependent M1 polarization with a typical M1 macrophage expression profile. Furthermore, in a study in syngeneic tumor-bearing mice with intact immune systems, a second-generation CAR-iMac has been shown to exert anti-tumor effects while maintaining M1 polarization and fully mobilizing T cells and NK cells within the immune microenvironment, thereby achieving the transformation of tumors from “cold” to “hot” phenotypes (131). Thus, CAR-M-based approaches show encouraging potential for applications in treatment of solid tumors. To date, the Food and Drug Administration has granted clinical trial approval for two CAR-M therapies, namely CT-0508 and MY-M11. Future studies should explore further how CAR-M-based agents improve the immunosuppressive microenvironment, as well as investigating how they are regulated by various microenvironmental components such as TAMs upon entering the TME.

In the context of macrophage-targeted therapies, it is essential to mention macrophage immune checkpoints, such as PD-L1/PD-L2, SIRPα, V-domain Ig suppressor of T cell activation (VISTA), T-cell immunoglobulin and mucin-domain containing-3 (TIM3), B7 homolog 4 (B7-H4), Triggering Receptor Expressed on Myeloid Cells 2 (TREM2) and so on. PD-L1/PD-L2 have been extensively discussed. VISTA is an immune checkpoint protein highly expressed in myeloid cells, which binds to PSGL-1 to inhibit T cell activation and promote IL-10 secretion (132). VISTA blockade synergistically enhances anti-tumor immunity when combined with PD-1 antibodies (133). JNJ-61610588 is a monoclonal antibody that specifically targets VISTA, Phase I trials have demonstrated its safety in solid tumors (NCT02671955). TIM3 induces T cell apoptosis through the Galectin-9/TIM3 axis (134). TIM3/PD-1 bispecific antibodies, such as AZD7789, are currently being evaluated in advanced non-small cell lung cancer for their dual regulation of TAMs and T cells (NCT04612751). B7-H4 may promote tumor immune evasion by inhibiting T cell proliferation and cytokine secretion (135). The Preclinical studies of B7-H4-CAR-T have shown its ability to specifically eliminate B7-H4+ TAMs and activate T cells (136). The anti-B7-H4 antibody (HS-20089) is currently recruiting patients with recurrent or metastatic ovarian cancer and endometrial cancer (NCT06014190). TREM2 suppresses anti-tumor immune responses by inhibiting T cell activation and NK cell function (137). AL002 is a TREM2 agonistic antibody that promotes TREM2 activation, activates microglia, and induces phagocytosis (138). Inhibiting TREM2 is an effective strategy to enhance anti-tumor T cell activity, and blocking TREM2 protein enhances the efficacy of PD-1 antibodies, completely eradicating tumors in mice (139). Immune checkpoint inhibitors are increasingly being used in combination with various other targeted therapies, such as poly ADP-ribose polymerase (PARP) inhibitors and PI3K inhibitors (140).

Apart from targeting macrophages, the targeting of other components in the TME has also received considerable attention. For instance, the monoclonal antibody fusion protein Simlukafusp alfa (also known as FAP-IL2v) targets fibroblast activation protein (FAP) in CAFs. It can localize to the tumor site with the FAP monoclonal antibody, and the IL-2 mutant can bind and regulate the IL-2Rβγ dimer on the surface of T cells in the TME, thereby activating the anti-tumor immune effect in the microenvironment. Combined with PD-L1 antibody, it showed partial response, but the targeting specificity was limited (NCT03386721). Mogamulizumab, targeting effector regulatory T cells, is an anti-CCR4 monoclonal antibody (141), which can reduce Treg infiltration and enhance anti-tumor immunity (NCT02301130). In a phase III clinical trial of melanoma, the indoleamine 2, 3-dioxygenase 1 (IDO1) inhibitor Epacadostat suggested that the combination with PD-1 inhibitor did not reach the primary endpoint, but subgroup analysis showed partial benefit (NCT02752074) (142). It can be found that targeting TME including TAMs tend to be combined with radiotherapy and chemotherapy or ICB immunotherapy in current clinical trials (see Table2).

Finally, a comprehensive understanding the influence of various components of the TME on TAMs necessitates consideration of tissue localization, especially regarding therapeutic regimens targeting TAMs. Studies have demonstrated that nanoparticles loaded with drugs capable of reprogramming macrophages from a pro-tumor M2 polarization state to an anti-tumor M1 state can enhance immunotherapy and improve overall therapeutic efficacy (143). However, the phenotype and function of TAMs may revert to the previous M2-like immunosuppressive state upon discontinuation of treatment with the repolarizing drugs (144). Furthermore, the ultimate state of macrophages is regulated by multiple factors within the TME and by various metabolic pathways intrinsic to TAMs. These factors contribute to the limitations of current drugs targeting macrophages and the challenge of maintaining therapeutic efficacy after drug cessation (107), Determining the optimal dosage and frequency of TAM-targeted therapy therefore remains a significant challenge, and identifying appropriate pathways to ensure both the efficacy and safety is also a critical area of ongoing research.

This review comprehensively describes the impact of different components of TME on TAMs. Due to space limitations, we have focused mainly on the cellular components of the TME. However, the influence of non-cellular components such as cytokines, metabolites, and the ECM on TAMs has also been partially covered. The non-cellular components of the tumor microenvironment are complex and variable, and further in-depth research is needed in the future. In conclusion, this review highlights the importance of fully understanding the impact of different components of the TME on TAMs in the development of targeted cancer therapies.
